# The Evolution of Blood Pressure Thresholds and Targets over Time: A Historical Review

**DOI:** 10.3390/medsci14020203

**Published:** 2026-04-17

**Authors:** Maria Elena Flacco, Flavia Minoia, Gabriele Brunini, Martina Rosticci, Matteo Fiore, Giancarlo Cicolini, Cecilia Acuti Martellucci, Claudio Borghi, Lamberto Manzoli

**Affiliations:** 1 Department of Environmental and Prevention Sciences, University of Ferrara, 44121 Ferrara, Italy; mariaelena.flacco@unife.it; 2 Department of Innovative Technologies in Medicine & Dentistry, ‘G. d’Annunzio’ University of Chieti-Pescara, 66100 Chieti, Italy; minoiaflavia@gmail.com (F.M.); g.cicolini@unich.it (G.C.); 3 Department of Medical and Surgical Sciences, University of Bologna, 40126 Bologna, Italy; gabriele.brunini@studio.unibo.it (G.B.); matteo.fiore7@studio.unibo.it (M.F.); c.acutimartellucci@unibo.it (C.A.M.); claudio.borghi@unibo.it (C.B.); 4 IRCCS Istituto Romagnolo per lo Studio dei Tumori (IRST) “Dino Amadori”, 47014 Meldola, Italy; martina.rosticci@irst.emr.it

**Keywords:** hypertension, blood pressure, guidelines, review

## Abstract

The definition of hypertension and the values of systolic and diastolic blood pressure (BP) that should be considered as therapeutic targets have changed over time and vary across scientific societies, which may generate uncertainty in the decision-making process among clinicians and patients. We traced the evolution and described the differences in all the 32 Clinical Practice Guidelines for the management of hypertension released by the following national and international scientific societies: World Health Organization—WHO; International Society of Hypertension—ISH; Joint National Committee on Prevention, Detection, Evaluation, and Treatment of High Blood Pressure—JNC; American Heart Association—AHA; American College of Cardiology—ACC; European Society of Cardiology—ESC; European Society of Hypertension—ESH; and UK National Institute for Health and Care Excellence—NICE. Throughout the decades, the BP values used for hypertension definition, treatment initiation, and targets to achieve started from SBP/DBP ≥ 160/95 mmHg, established at the end of the 70s, progressively decreased, and were differentiated by individual cardiovascular risk. In the last decade, a divergent approach emerged across scientific societies: while WHO/ISH and NICE recommended thresholds and targets for the general population at SBP/DBP < 140/90 mmHg, ESH/ESC and ACC/AHA guidelines further and markedly reduced both BP threshold values and therapeutic targets, recommending as ideal SBP/DBP values < 130/80 mmHg and encouraging an SBP < 120 mmHg. Discrepancies also emerged in the assessment of the quality of the evidence: although the methodological approaches largely improved over time and across all the institutions assessed, various degrees of incompleteness on the adopted scales were reported, and potentially conflicting situations emerged, particularly when weaker evidence was used to build strong recommendations. Although some degree of discrepancy among guidelines is expected, some of the differences are large and can lead to widely different approaches in the management of BP control. A standardization of the methodology and interpretation of the evidence supporting the guidelines may help to reduce the variability in order to provide the best possible guidance for clinical practice and patient health.

## 1. Introduction

According to recent estimates, 1.4 billion adults aged 30–79 years are currently living with a diagnosis of hypertension [1], which is a major risk factor for several life-threatening cerebrovascular and cardiovascular diseases [2]. For decades, several approaches have been recommended to lower blood pressure (BP) and maintain such an effect over time, either through lifestyle modifications and/or pharmacological treatment [3]. In the process, considerable attention has been paid to the identification of the values of systolic and diastolic BP that should be considered “optimal”, both in the general population and in specific at-risk subgroups, and recommended to both physicians and patients as “targets” to achieve [4,5,6]. Along with the evolving knowledge of cardio- and cerebrovascular diseases pathophysiology, the definition of these targets and, subsequently, of what is considered hypertension, has varied multiple times [5,7,8], as reported by the various evolving BP guidelines [5], with remarkable implications on the clinical choices and everyday management of hypertensive patients by healthcare providers [9,10].

The present historical review aims to trace and describe the evolution of the thresholds and target values for hypertension, from the earliest guideline statements to the most recent international recommendations.

## 2. Materials and Methods

### 2.1. Literature Search and Inclusion Criteria

MedLine and Scopus databases were initially searched to identify all hypertension guidelines, published in English, from the earliest available editions to the most recent versions, up to 31 December 2025.

National and international Clinical Practice Guidelines (CPGs) that comprised statements on the management of hypertension, issued by major professional and scientific societies, were included. Specifically, we focused on the guidelines published throughout the years by the following organizations, which have been considered as a reference in multiple countries over time [11]:

(a) World Health Organization (WHO) and International Society of Hypertension (ISH);
(b) US National Institute of Health/Heart, Lung and Blood Institute—Joint National Committee on Prevention, Detection, Evaluation, and Treatment of High Blood Pressure (JNC);
(c) American Heart Association (AHA);
(d) American College of Cardiology (ACC);
(e) European Society of Cardiology (ESC);
(f) European Society of Hypertension (ESH);
(g) UK National Institute for Health and Care Excellence (NICE).

Guidelines solely focused on the pharmacological treatment of hypertensive individuals, as well as those specifically dealing with the management of hypertension among special populations and/or in the presence of comorbid conditions (e.g., obesity, diabetes, dyslipidemia, pregnancy), were excluded.

The bibliographic search was performed by two investigators independently (M.E.F. and G.B.), and the last search update was performed on 21 January 2026. The following keywords: “blood pressure”, “hypertension”, “high blood pressure”, “hyperpiesis”, and “guideline” were used to build specific search strings, adapted to each database: (blood pressure[Title/Abstract]) OR (BP[Title/Abstract]) OR (hypertension[Title/Abstract]) OR (high blood pressure[Title/Abstract]) OR (hyperpiesis[Title/Abstract])) AND (guideline*[Title/Abstract]) for Medline; (TITLE-ABS-KEY(blood pressure) OR TITLE-ABS-KEY (bp) OR TITLE-ABS-KEY (hypertension) OR TITLE-ABS-KEY (high blood pressure) OR TITLE-ABS-KEY (hyperpiesis) AND TITLE-ABS-KEY (guideline*)) for Scopus. The reference lists of the retrieved articles were also screened for additional pertinent papers. During the process of literature search, efforts were made to identify potentially relevant publications not published in Medline- or Scopus-indexed journals. Accordingly, an extra search was carried out on Google using “blood pressure”, “hypertension”, and “guideline” as keywords. Moreover, the official websites of the seven above-mentioned scientific organizations were screened in order to detect all the documents or guideline versions made available on each specific website.

In order to trace back the evolution over time of each CPG, the introduction sections of the latest available editions were first screened to detect any mentioned earlier version, which was also referenced in the bibliographic section. Multiple versions of the same CPG, either when published in different scientific journals or available on different websites in the case of joint statements, were identified by comparing the title and the abstract/introduction of each document. When insufficient information was available, additional bibliographic sources, recommended by expert cardiologists, were checked to confirm the exact publication sequence.

### 2.2. Data Extraction

For each included guideline and for each edition, we extracted the following information: (a) diagnostic criteria adopted to define hypertension (diagnostic thresholds); (b) blood pressure (BP) values for which non-pharmacological treatments are recommended; (c) systolic (SBP) and/or diastolic BP (DBP) values for which pharmacological treatment is indicated (treatment thresholds); (d) target BP values to be reached and maintained (treatment targets); (d) any threshold and target value for high-risk individuals, as well as age-specific indications, when available; (e) level of evidence/strength of each recommendation, if explicitly stated. All extracted data were reported in a separate Excel spreadsheet to generate a summary table for each scientific organization over the years. When possible, the evolution over time of each threshold and target BP value was visually plotted into a graph.

## 3. Results

**Figure 1:**
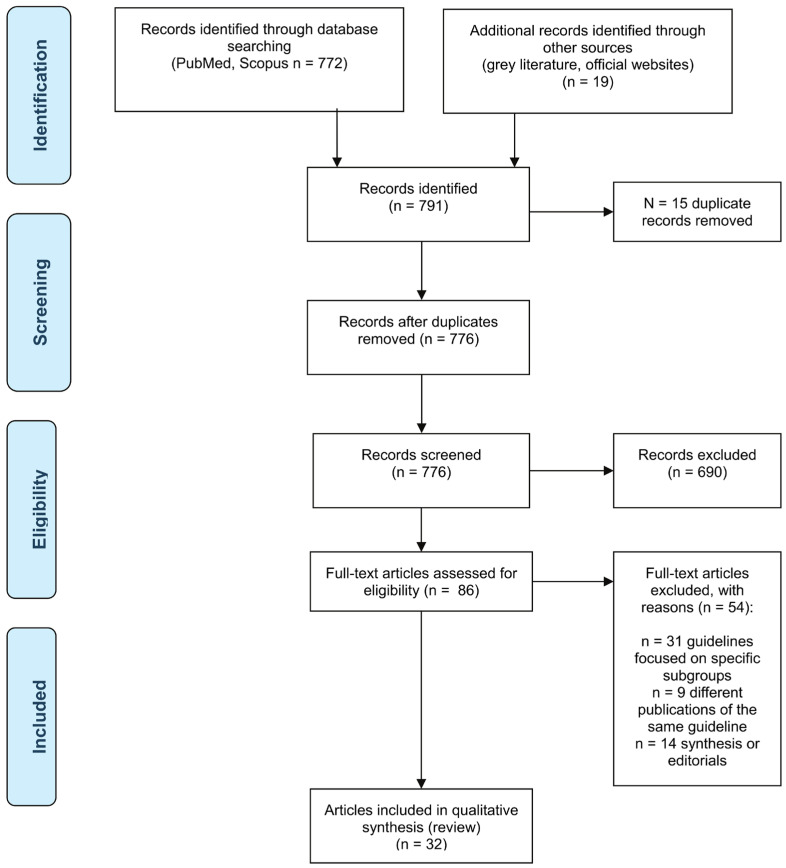
PRISMA flow diagram for the article selection process.

The initial search yielded a total of 791 items; after the title/abstract screening process, 86 publications were further assessed for eligibility, and 32 were finally included (Figure 1). Of these, 10 reports were issued by US-based institutions (*n* = 2 by the ACC/AHA [12,13]; *n* = 8 by the JNC [14,15,16,17,18,19,20,21]); 6 documents were released by European societies, either jointly published by ESC/ESH (*n* = 4) [22,23,24,25] or released as separate guidelines by ESH [26] and by ESC [6], respectively; 9 guidelines were published by global institutions, such as the WHO [27], the ISH [28], or jointly [29,30,31,32,33,34,35], 7 by British professional organizations (BHS and NICE) [36,37,38,39,40,41,42]. Their main characteristics, including the diagnostic criteria for hypertension, the thresholds for pharmaceutical treatment initiation, and the recommended BP targets, have been listed in Table 1, Table 2, Table 3 and Table 4.

### 3.1. Hypertension Thresholds and Targets Before the Implementation of the Clinical Guidelines

In the first decades of the 20th century, hypertension was not considered a cardiovascular risk factor in itself but rather a symptom, suggestive of an underlying condition. The diagnosis was made when SBP exceeded 150 mmHg for at least two weeks [46], or when the mean arterial pressure was >135 mmHg [48], and no increase in SBP was expected after 60 years of age due to a less strenuous lifestyle and a weakening of the cardiac muscle [49].

It was only after the 40s, and particularly after the death of US President Franklin Delano Roosevelt in 1948, that hypertension started conveying wide interest among the scientific community [50]. From these years and still in the following decades, its diagnosis relied primarily on DBP, defined as a resting BP ≥ 100 mmHg [50]. This cut-off was still widely accepted by the scientific community in the 1970s, when the introduction of the first thiazide diuretics led to a substantial improvement in the therapeutic approach to essential hypertension, and most research focused on the identification of the best DBP threshold value to initiate pharmacological treatment. In 1976, this threshold was finally confirmed at a DBP ≥ 100 mmHg by the British Cardiac Society [51].

In 1977, as a result of the efforts of the National High Blood Pressure Education Program and the National Heart, Lung, and Blood Institute, the Joint National Committee on Prevention, Detection, Evaluation, and Treatment of High Blood Pressure (or JNC) issued the first official antihypertensive guideline [14].

### 3.2. US Guidelines: JNC and ACC/AHA

In the US, the first official guidelines on the management of hypertension were released by the JNC within the framework of the National Institute of Health/Heart, Lung, and Blood Institute (NIH/NHLB). As mentioned, the inaugural edition—the so-called JNC 1—was published in 1977 [14]. In this report, as well as in the first three that followed (JNC 2, 3, and 4 in 1980, 1984, and 1988, respectively [15,16,17]), a diastolic blood pressure (DBP) ≥ 90 mmHg served as the primary criterion to define hypertension, while the presence of a systolic BP ≥ 140 mmHg was only taken as an ancillary parameter or to strictly define the condition of isolated hypertension. Starting from the JNC 5 (published in 1993) onwards, a combined systolic–diastolic criterion, based upon an SBP ≥ 140 mmHg, in addition to (or as an alternative to) a DBP ≥ 90 mmHg, was introduced to identify hypertensive individuals [18]. Also, in the JNC5, a new class of “high-normal blood pressure” was established to define a category of subjects (with SBP ranging between 130 and 139 mmHg and/or DBP between 85 and 89 mmHg) showing an increased risk of developing hypertension and of experiencing nonfatal and fatal cardiovascular events compared with otherwise similar persons with lower BP levels [18]. This borderline subgroup was further modified in the JNC 7 (published in 2003), where the category of “high-normal” BP was replaced with the broader condition of “pre-hypertension” to include individuals with an SBP of 120 to 139 mmHg or a DBP of 80 to 89 mmHg [20], with unchanged threshold values for hypertension (SBP ≥ 140 mmHg and/or DBP ≥ 90 mmHg).

Along with the variations in the diagnostic criteria during the years, there was a change also in the threshold BP values to initiate pharmacological treatment (PT), as well as in the target BP to be achieved: if in the first two JNC guidelines the PT threshold was set at a DBP ≥ 105 mmHg, it fell to DBP ≥ 95 mmHg in the third and fourth reports. Since the fifth JNC guideline onwards, the coexistence of an SBP > 140 mmHg and a DBP > 90 mmHg was the new, combined threshold for therapy start, and, from the sixth JNC [19], the presence of only one of the two was deemed sufficient (SBP > 140 mmHg or DBP > 90 mmHg). The last report of the series was the JNC 8 (published in 2014) [21], after which the guidelines jointly released by the American College of Cardiology/American Heart Association (ACC/AHA) became the primary reference framework for hypertension management in the US. In JNC 8, no specific target for the diagnosis of hypertension was set; however, the threshold for therapy start was broadened to include an SBP ≥ 140 mmHg (or ≥150 mmHg for the elderly) or a DBP ≥ 90 mmHg.

Parallel to the BP thresholds, the target BP levels also underwent a progressive shift: initially (in JNC 1 up to JNC 4), a DBP < 90 mmHg was the only target value; later, from JNC 5 onwards, this definition was revised to include both an SBP < 140 mmHg and a DBP < 90 mmHg; and finally, lower, additional targets for specific categories were set: ≤130/85 mmHg for individuals >60 years (in JNC 5) and <130/80 mmHg in the presence of diabetes or kidney disease (in JNC 7).

As mentioned, the last JNC report was issued in 2014, and from 2017, with the first published guideline, ACC/AHA reports became the primary reference framework for hypertension management in the US. Supported by evidence primarily from the Systolic Blood Pressure Intervention Trial (SPRINT) [44], these guidelines set the new diagnostic criteria at SBP ≥ 130 mmHg or DBP ≥ 80 mmHg [13]; also, the ACC/AHA 2017 guidelines retained from earlier JNC documents the category of pre-hypertension, now updated to include SBP values between 120 and 129 mmHg and DBP values < 80 mmHg [13]. Additionally, the ACC/AHA guidelines introduced a shift in the criteria for treatment initiation: while earlier documents recommended pharmacological treatment primarily at ≥140/90 mmHg, the 2017 report introduced a tighter control, defined upon individual cardiovascular risk. In particular, the risk stratification was based upon the evaluation of BP levels, presence of comorbidities, and metabolic risk factors to compute for each individual a 10-year risk of incident CVD (ASCVD). In this scenario, a risk-based treatment initiation was set at: (a) ≥130/80 mmHg for individuals with established cardiovascular disease or ≥10% ASCVD risk and (b) ≥140/90 mmHg for individuals with <10% ASCVD risk. In all cases, the recommended target values were <130/80 mmHg.

The latest update of the ACC/AHA guidelines [12], while leaving diagnostic criteria unchanged, adopted a more stringent approach to define the risk of developing a CVD within 10 years. In particular, the updated cut-off was set at 7.5%, and, at any BP level, the presence of a 10-year risk ≥ 7.5% alone was an indication to start therapy, particularly in the presence of diabetes or renal damage. In this updated paradigm, the ideal (and encouraged) BP targets for SBP/DBP were lowered to <120/80 mmHg for all hypertensive individuals, irrespective of the 10-year CVD risk [12].

In earlier JNC reports, there was no explicit mention of the quality of scientific evidence or the strength of the recommendations. Only in the last JNC report [21], for the first time in US-based guidelines, was each recommendation assigned a specific grade based upon the quality (the design) of the supporting evidence, rated as High, Medium, or Low (Table 1).

Both reports released by ACC/AHA used an internal tool to score the quality of the evidence based upon study design (from A—multiple large RCTs and meta-analyses of RCTs—to C—expert opinion). The panel adopted an internal score also to classify the strength of each recommendation (from Class 1—Strong, when benefits largely outweigh risks, to Class 3—No benefit or harmful statement). Notably, the panel reported that the class of recommendations and quality of evidence were mutually independent; thus, throughout the document, any level of evidence could be paired with statements of any strength.

### 3.3. International Guidelines: WHO and ISH

The first WHO report on the diagnosis and management of hypertension was released in 1978 [27], just a year after the first JNC guideline. Hypertension was initially defined as “SBP ≥ 160 mmHg and/or DBP ≥ 95 mmHg”, and treatment was recommended at SBP/DBP values > 140/90 mmHg, respectively, with an opposite target at ≤140/90 mmHg. The use of non-pharmaceutical interventions (NPI) and medications was left at the physician’s discretion.

In 1983, the WHO and ISH published the first joint guideline, in which the definition of hypertension was lowered to DBP ≥ 90 mmHg (regardless of SBP values), the therapy initiation was set at DBP > 95 for 3 months, and the BP target was set at <90 mmHg [29]. These criteria remained quite similar in the 1986 and 1989 versions of the joint WHO/ISH reports [30,31], but the 1993 revision substantially lowered the BP values to diagnose hypertension (SBP ≥ 140 mmHg and/or DBP ≥ 90 mmHg) [32]. These values remained stable in all subsequent editions and are still the same as the last WHO/ISH guideline, published in 2021 [35]. Similarly, the SBP and DBP targets that were identified in 1993 (<140 mmHg and <90 mmHg, respectively) still hold currently, although an additional target of SBP < 130 mmHg was currently recommended in patients with or at high risk for cardiovascular diseases [35]. Indeed, starting from the 1999 edition onwards [33], the primary determinant for treatment initiation gradually shifted from BP levels alone to include an individual risk stratification based upon age, gender, metabolic risk factors, history of cardiovascular disease, and organ damage. In this new paradigm, pharmacological therapy was recommended for individuals with SBP/DBP ≥ 140/90 mmHg at moderate or high cardiovascular risk, while lower-risk individuals were managed primarily through lifestyle interventions.

In all the earlier guidelines, the scientific evidence was not rated, and the strength of the recommendations was not reported. This scenario remained unchanged up to the 2020 ISH report, in which each statement was classified as “Essential” or “Optimal”, although no explicit reference to the supporting evidence was made [28]. The approach changed with the 2021 edition of the WHO/ISH joint guidelines, when a score was assigned to each recommendation (strong vs. weak or conditional) based upon a rating of the scientific evidence that was performed through the GRADE approach, which identifies strong-, moderate-, low-, and very low-quality evidence [52].

### 3.4. European Guidelines: ESH and ESC

Until 2003, the European authorities relied on WHO/ISH guidelines for official recommendations on hypertension management. From 2003 up to 2018, ESH and ESC published joint guidelines in order to address the peculiar features of the European population, characterized by a high proportion of elderly individuals, limited racial disparities, and generally elevated socioeconomic conditions, including a wide availability of healthcare resources [22,23,24,25]. After 2018, ESH and ESC published separate guidelines in 2023 and 2024, respectively [6,26].

Across successive editions, the European guidelines showed a high degree of continuity in the definition of hypertension, consistently maintaining a diagnostic threshold of SBP ≥ 140 mmHg and/or DBP ≥ 90 mmHg, a threshold that is currently maintained. Along with the stability in the diagnostic criteria, in all editions of the European guidelines, the decision to initiate antihypertensive therapy was broadly based upon a risk-stratified strategy, where the individual risk of CVD was the primary parameter for drug therapy, integrated, as a secondary source of information, with BP levels, irrespective of patients’ age. The risk stratification was based upon the SCORE Chart, which, considering several risk factors and each patient’s clinical history, stratifies the 10-year risk of developing a CVD into low, moderate, high, and very high [52]. Since the 2003 edition, up to the 2023 ESH guideline, treatment initiation SBP/DBP thresholds were set at ≥140/90 mmHg, in particular for patients with very high/high-to-moderate cardiovascular risk or in the presence of CVD, diabetes, or renal disease. A tighter approach was chosen in the last available European guideline, released in 2024 by the sole ESC [6]. Here, antihypertensive therapy was recommended for all the individuals with a cardiovascular risk at least borderline (i.e., ≥5%) if BP levels are ≥120 mmHg (SBP) and/or ≥70 mmHg (DBP).

As regards the therapeutic targets, they were set at an SBP < 140 mmHg and a DBP < 90 mmHg for all individuals in the first, 2003 edition of the ESC/ESH guidelines (with the exclusion of diabetic patients and those with a history of CVD, stroke, or proteinuria, with targets < 130/80 mmHg). These values remained stable until the 2018 guideline, which recommended lower targets (SBP/DBP < 130/80 mmHg, respectively), which have also been maintained in the newest, 2023 and 2024 guidelines.

With the exception of the two earlier documents (ESC/ESH 2003 [22] and 2007 [24]), all the European guidelines adopted a predefined methodology to score the evidence and the strength of the statements, explicitly described in the publications and unchanged throughout the years: the quality of the available evidence (rated from A to C according to the study design, with multiple RCTs and meta-analyses on top) was used to classify the strength of each recommendation, classified as I (strongest) to III (weakest).

### 3.5. UK Guidelines: BHS and NICE

The first official English guideline on hypertension was released by BHS in 1989. In line with other reports published in the same years, hypertension was diagnosed relying only upon DBP values, which were set at ≥100 mmHg. The same parameter was also used as a threshold for pharmacological treatment, with a recommended target of <100 mmHg [36]. In the third edition, published in 1999, the diagnostic criteria were modified to include also SBP values ≥ 140 mmHg, in combination with DBP, now set at ≥90 mmHg. The treatment thresholds were also refined, including SBP/DBP ≥ 160/100 mmHg for individuals without risk factors and ≥140/90 mmHg for those with diabetes, organ damage, a history of CVD, or a 10-year risk of developing a CVD ≥ 15% [40]. Since 2004, when the first guideline released by the NICE was published [42], up to the latest edition (2023) [38], the diagnostic criteria for hypertension (SBP ≥ 140 mmHg and/or DBP ≥ 90 mmHg) and the BP thresholds for medication initiation (SBP/DBP ≥ 160/100 mmHg) did not change.

As regards the treatment targets, they remained stable after 1993 at SBP < 140 mmHg and DBP < 90 mmHg for the general hypertensive individuals, although in the last guideline, published in 2019 and updated in 2023, specific target values were established for elderly subjects (<150/90 mmHg) or for individuals with diabetes or renal damage (<130/80 mmHg) [38].

Starting from 1999 BHS III onwards, all the published British documents reported a classification system for the quality of the evidence and the strength of the recommendations. All guidelines adopted a standardized system (initially the North of England Evidence-Based Guidelines System [53] and later the Agency for Healthcare Research and Quality Classification System [37]) to classify each study quality based upon study design, rated on a scale from I—evidence available from meta-analyses of RCTs to IV—evidence available from expert opinion or committees. This classification determined different levels of strength of the recommendations, from Level A—based upon category I—to Level D—based upon category IV. Interestingly, in the last three NICE documents, although the adoption of this system was declared, the reported recommendations were not assigned a specific score for their strength.

## 4. Discussion

The present review of some of the most commonly followed guidelines worldwide shows a slowly but continuously evolving scenario pertaining to hypertension definition and management, which may be roughly divided into five distinct phases.

First, before the introduction of the guidelines, hypertension was treated only when clinically manifest (being considered a symptom suggestive of an underlying condition) and was identified with very high SBP values (>150 mmHg for at least two weeks). While non-pharmacological approaches had been recommended since the 1910s, it was only in the 1960s that new drugs were introduced for asymptomatic hypertensive individuals [54].

In the second phase (1977–1992), when the first official guidelines were released, the consolidation of the evidence regarding hypertension led to the definition of precise therapeutic targets and a change in treatment indications, initially based on DBP alone and subsequently on both SBP and DBP. The BP values to define a patient as hypertensive, however, were still high as compared with current parameters (e.g., the 1978 WHO diagnostic criteria indicated levels of SBP/DBP ≥ 160/95 mmHg).

In the third phase (1993–2004), the therapeutic thresholds decreased, and both thresholds and targets were refined based on the individual cardiovascular risk, in addition to BP levels. In this phase, there was a general consensus across guidelines on the therapeutic targets to be reached by individuals without risk factors (<140/90 mmHg) and with risk factors, such as coronary heart disease, chronic kidney disease, diabetes, or high risk of future cardiovascular diseases (<130/80 mmHg).

In the fourth phase (2005–2017), BP thresholds and targets remained stable, and in two cases they were even increased (ESC/ESH 2013 [23] and JNC 8 [21] guidelines, which raised BP targets and thresholds by 10 mmHg for high-risk individuals). This phase was also characterized by some changes in the entities responsible for issuing guidelines: NICE replaced BHS and became the only official English body entitled to release guidelines, and the joint ACC/AHA took over the previous US NHLBI and the JNC.

**Figure 2:**
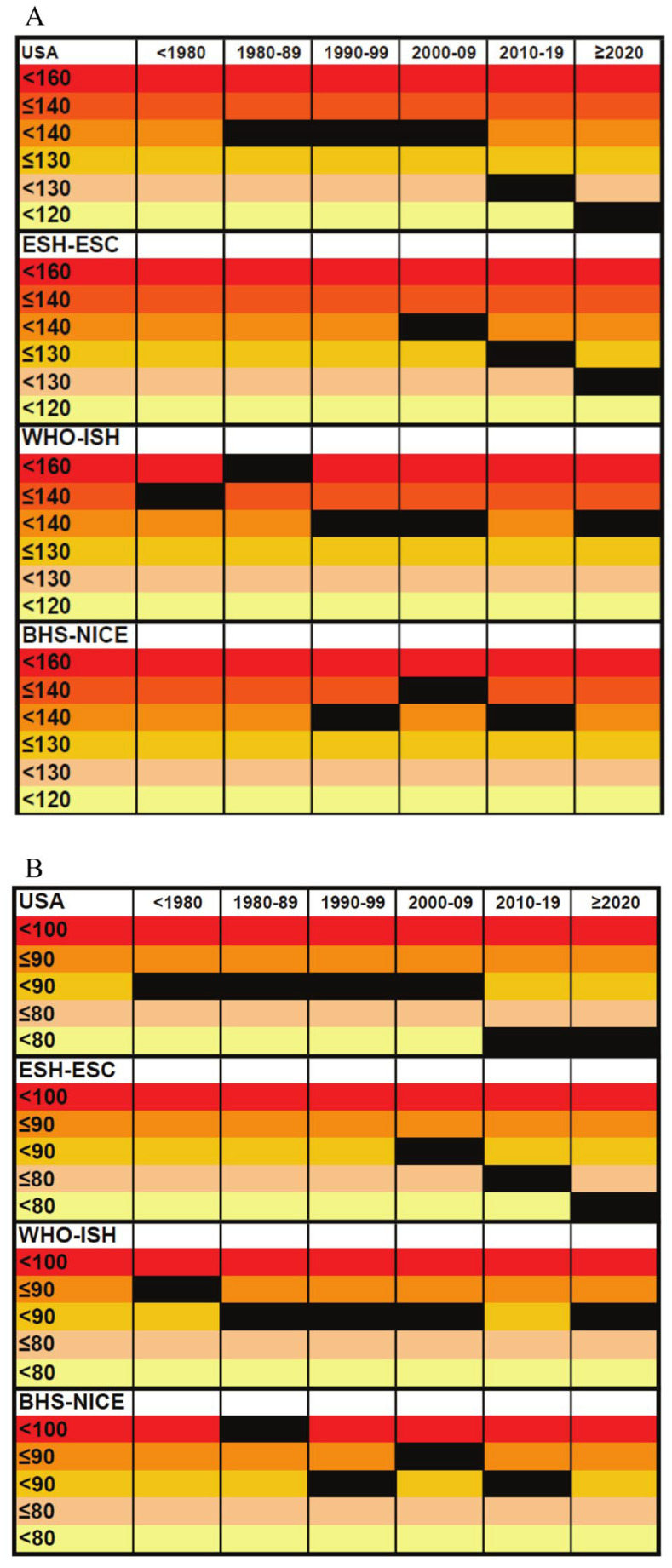
Trend over time of the therapeutic targets recommended by each guideline on systolic (**A**) and diastolic (**B**) blood pressure. USA: Guidelines from the US National Institute of Health/Heart, Lung and Blood Institute—Joint National Committee on Prevention, Detection, Evaluation, and Treatment of High Blood Pressure, and/or the American Heart Association/American College of Cardiology. ESH-ESC: European Society of Hypertension and/or European Society of Cardiology. WHO-ISH: World Health Organization and/or International Society of Hypertension. BHS-NICE: British Hypertension Society and/or National Institute for Health and Care Excellence.

The fifth and last phase (2017–2025) has been characterized by a differentiation of the guidelines (Figure 2): while WHO/ISH and NICE recommended thresholds and targets that, for the general (low-risk) population, remained unchanged at SBP/DBP < 140/90 mmHg, ESH/ESC and ACC/AHA guidelines further and markedly reduced both BP threshold values and therapeutic targets: both European and US latest reports recommended as ideal SBP/DBP therapeutic targets < 130/80 mmHg, encouraging an SBP < 120 mmHg. The currently accepted definitions, thresholds, and targets, and grading approaches, issued by each of the included organizations, have been reported in Table 5.

Several potential explanations, not mutually exclusive, have been called into question to explain the reported discrepancies across institutions. First, they reflect a scenario of ever-evolving, sometimes conflicting evidence. Indeed, the lower BP targets recommended by European and US-based institutions as compared to international and UK-based ones were largely influenced by findings from the 2015 SPRINT, in which lower rates of fatal and non-fatal cardiovascular events were observed among subjects treated to a target SBP < 120 mmHg versus <140 mmHg [44]. Notably, these findings were not taken into account by the NICE committee, which expressed concerns regarding (a) the methodology of BP measurement adopted by the trialists, not applicable to routine UK clinical practice; (b) the heterogeneity of the study population; and (c) the limited availability of long-term safety data [55]. Additionally, a 2020 Cochrane review showing no reduction in total mortality with BP targets <135/85 and the results of the HYVET trial recommending less stringent thresholds and treatment targets for the elderly may contribute to explaining the variations in targets across CPGs [47,56]. Second, the observed discrepancies may reflect the complex trade-off between experimental evidence gathered from RCTs and targets achievable in local contexts, in everyday clinical practice [8]. Third, differences across organizations in committee composition, process of guidelines generation, and aims may contribute to the existing variation [57]. As an example, while NICE produces government-funded CPGs based upon a combination of clinical evidence and cost-effectiveness analyses, intended for both primary and secondary care physicians, as well as for health economists, both ACC/AHA and ESC/ESH guidelines are mainly focused on the clinical management of US and European individuals across a wide set of countries, healthcare systems, and clinical conditions [57].

In conclusion, the present review identified a total of 32 national and international guidelines for the diagnosis and management of hypertension, which have undergone numerous changes over the last century. Throughout the years, all professional societies have promoted a change in the definition of hypertension, lowering the BP threshold value for diagnosis, to initiate antihypertensive treatment, and the relative therapeutic targets. On one side, this approach, following the emergence of new evidence, reflects the aim of extending treatment benefits to patients with early disease, in line with the well-known Geoffrey Rose’s Prevention Paradox [58]. On the other side, with many more patients becoming eligible for pharmacological therapy or for more severe treatment schemes, the overall costs of hypertension management inevitably rise, as well as the proportion of subjects who are unable to achieve the proposed targets [8]. An in-depth evaluation of the cost–benefit of the threshold lowering is far beyond the scope of the present review, but two critical issues are apparent and deserve to be mentioned: first, the current guidelines are markedly different on the therapeutic targets to be achieved by the general, low-risk population. Although some degree of discrepancy among guidelines is expected, given the potential differences in weighting and interpretation of the available research findings by each guideline’s writing committee [8,59], these discrepancies generate uncertainty in the decision-making process among clinicians, especially when national recommendations diverge from international ones [60]. Additionally, in the current scenario of health literacy, where many patients seek health advice online [61], the availability of contrasting recommendations is likely to generate some confusion and distrust towards divergent clinicians’ judgments [9].

Second, discrepancies also emerged in the completeness of information regarding the process of quality evaluation of the supporting evidence. The reported methodological approaches and the tools adopted to score the scientific literature largely varied across professional societies and, within the same body, throughout the years. Additionally, not all the quality evaluations, when performed, were used to grade the strength of the recommendations, which led to some “strong” recommendations based upon “expert opinion”. Although it may represent a violation of the classical EBM framework [62], two issues should be considered. First, this finding may reflect a lack of robust, uniform evidence in certain research areas, highlighting the need for further clinical research in some specific subfields of hypertension diagnosis and/or management [63]. Second, in the need of additional clinical guidance, when extensive evidence is lacking to support physicians’ decision-making process, the production of CPGs cannot rely only on high-level clinical evidence, and expert opinion, personal judgement, or organizational preferences can play an important role [8,63].

The present review has some limitations that must be considered. First, only the CPGs issued by selected scientific organizations and institutions were included (although taken as pivotal for multiple countries over time [11]). Second, we did not include guidelines that encompass recommendations for multiple conditions potentially coexisting with hypertension (such as obesity, diabetes, or pregnancy). Accordingly, the present review cannot be considered a comprehensive evaluation of the management of hypertension in a global scenario. Third, although the bibliographic search was performed across multiple databases, we might have missed some specific documents focused on particular settings (such as frail settings); thus, the adopted methodology may be biased towards including guidelines conceived for a high-income scenario. However, the present review strictly focused on the definitions of treatment thresholds and BP targets, which are much less influenced by socio-economic conditions than pharmacological management [64], whose description was beyond the scope of the present review.

Given the huge number of people affected [1], further, in-depth analyses focused on the methods and the evidence supporting the different available guidelines are required to help reduce the observed discrepancies and provide the best possible guidance for clinical practice and patient health.

## Figures and Tables

**Table 1:** US National Institute of Health/Heart, Lung and Blood Institute—Joint National Committee on Prevention, Detection, Evaluation, and Treatment of High Blood Pressure (NHLBI—JNC) and American Heart Association/American College of Cardiology (ACC/AHA)—Overview of the included Clinical Practice Guidelines (CPGs).

Year and Edition	Ref.	HBP Definition, in mmHg	NPI Before PT	Treatment Threshold, in mmHg	Treatment Target, in mmHg	High-Risk Condition(s)	Quality and Strength of the Recommendations [Supporting Evidence]
1977 JNC 1 (NHLBI)	[14]	DBP ≥ 90	3–6 months	≥105 DBP; 90–105 DBP for HR	<90 DBP	High SBP; familiar history of complications due to high BP; organ damage; male sex; smoke; DM; high cholesterol	NR
1980 JNC 2 (NHLBI)	[15]	DBP ≥ 90 with: 90–104 mild; 105–114 moderate; ≥115 severe	Recommended for uncomplicated mild HBP	≥105 DBP; 90–105 DBP for HR	<90 DBP	High SBP; familiar history of complications due to high BP; organ damage; smoke; DM; high cholesterol	NR
1984 JNC 3 (NHLBI)	[16]	DBP ≥ 90 or SBP ≥ 140 in 2 or more measurements (DBP 85–89 high-normal)	Recommended for those with (a) DBP 90–94 mmHg and no RF; (b) isolated SBP ≥ 160 mmHg	≥95 DBP	<90 DBP; <160 SBP	Organ damage; male sex; smoke; DM; high cholesterol	NR
1988 JNC 4 (NHLBI)	[17]	DBP ≥ 90 or SBP ≥ 140 in 2 or more measurements	Recommended for those with (a) DBP 90–94 mmHg and no HR; (b) isolated SBP ≥ 160 mmHg	≥95 DBP; 90–94 DBP for HR	<140/90 BP; <160 if isolated HBP	Organ damage; male sex; smoke; DM; high cholesterol	NR
1993 JNC 5 (NHLBI)	[18]	SBP ≥ 140 and/or DBP ≥ 90 (high-normal 130–139 SBP; 85–89 DBP)	3–6 months	SBP > 140 AND or DBP > 90 (stage I HBP)	<140 SBP AND <90 DBP (ideally ≤130/85 for older individuals)	Organ damage; established CVD; CKD; dyslipidemia; smoke; physical inactivity; obesity	NR
1997 JNC 6 (NHLBI)	[19]	SBP ≥ 140 or DBP ≥ 90 (high-normal 130–139 SBP OR 85–89 DBP)	12 months for stage I HBP (140–159 SBP or 90–99 DBP) and no RF; 6 months for stage I HBP and 1 RF	BP > 140 or DBP > 90	<140 SBP and <90 DBP (optimal BP for CV risk is <120/80)	Risk stratification based upon BP levels plus presence/absence of organ damage, smoke, dyslipidemia, DM	Explicit mention to the strength of supporting evidence, and to grade it according to study design. No details provided on the strength of the recommendations
2003 JNC 7 (NHLBI)	[20]	SBP ≥ 140 or DBP ≥ 90 (stage I: SBP 140–159 or DBP 90–99; stage II: SBP ≥ 160 or DBP ≥ 100); pre-HBP: SBP 120–139 OR DBP 80–89	120–139 SBP or 80–89 DBP if no RF is present (unclear the duration of NPI)	BP > 140 or DBP > 90	<140 SBP and <90 DBP in the absence of RF; <130/80 in the presence of DM or CKD; <120/80 when in pre-HBP	Risk stratification based upon BP levels plus presence/absence of organ damage, smoke, dyslipidemia, DM	Explicit mention to the strength of supporting evidence, and to grade it according to study design. No details provided on the strength of the recommendations
2014 JNC8 (NHLBI)	[21]	Not specifically addressed, only thresholds for PT defined	Not specifically addressed	≥60 y: SBP ≥ 150 or DBP ≥ 90 (**grade A**); <60 y: DBP ≥ 90 (**grade A** only for 30–59 y); SBP ≥ 140 (**grade E**); all ages, CKD and/or DM: SBP ≥ 140 OR DBP ≥ 90 (**grade E**)	≥60 y: SBP < 150/DBP < 90; <60 y: SBP < 140/DBP < 90; CKD ± DM: SBP < 140/DBP < 90	Risk stratification based upon BP levels plus age; DM; CKD	Systematic literature review restricted to RCTs and m-a of RCTs; quality scored in High, Medium, and Low. Strength of recommendation grading system developed by the National Heart, Lung, and Blood Institute’s (NHLBI’s) Evidence-Based Methodology Lead
2017 ACC/AHA	[13]	SBP ≥ 130 or DBP ≥ 80 (stage I: SBP 130–139 or DBP 80–89; stage II: SBP ≥ 140 or DBP ≥ 90); pre-HBP: SBP 120–129 and DBP < 80; masked HBP: NBP OM; HBP out of OM; white coat HBP: HBP OM; NBP out of OM. White coat HBP: monitor to detect transition to HBP; if 120–129 or 75–79: monitor for masked HBP	3 months for pre-HBP, masked HBP and white coat HBP; 3–6 months for stage I HBP and ASCVD < 10%	Primary prevention: (a) ASCVD < 10% plus SBP ≥ 140 or DBP ≥ 90; (b) ASCVD ≥ 10% plus SBP ≥ 130 or DBP ≥ 80. Secondary prevention: SBP ≥ 130 or DBP ≥ 80, and clinical CVD	<130/80 (recommended for ASCVD ≥ 10% or clinical CVD; reasonable for ASCVD < 10%)	Risk stratification based upon: clinical CVD history, BP levels, plus 10-year risk of incident CVD computed using the ACC/AHA Pooled Cohort Equations (atherosclerotic CVD risk—ASCVD—estimator)	Systematic literature review to include all the available evidence, rated through LoE A-E. LoE and strength of the recommendation (COR I–III) are determined independently: any CoR can be associated with any LoE. Feasible to score the strength of each recommendation [SPRINT; ACCORD trial]
2025 ACC/AHA	[12]	SBP ≥ 130 or DBP ≥ 80 (stage I: SBP 130–139 or DBP 80–89; stage II: SBP ≥ 140 or DBP ≥ 90); pre-HBP: SBP 120–129 and DBP < 80	3–6 months in case of stage I HBP plus: no history of CVD, no DM, no CKD, PREVENT CVD risk < 7.5%	Primary prevention: (a) failure of NPI with stage I HBP, no DM or CKD, PREVENT CVD risk < 7.5%; (b) stage I HBP plus DM or CKD or PREVENT CVD risk ≥ 7.5%. Secondary prevention: SBP ≥ 130 or DBP ≥ 80, and clinical CVD. SBP ≥ 140 or DBP ≥ 90 irrespective of clinical CVD and CVD risk	At least < 130 SBP, encouraged < 120 SBP; < 80 DBP (recommended for all HBP, irrespective of increased PREVENT CVD ≥ 7.5%)	Risk stratification based upon: clinical CVD history, BP levels, plus 10-year risk of incident CVD computed using the ACC/AHA PREVENT CVD Risk estimator	Systematic literature review to include all the available evidence, rated through LoE A-E. LoE and strength of the recommendation (COR I-III) are determined independently: any CoR can be associated with any LoE. Feasible to score the strength of each recommendation

HBP: hypertension; DBP: diastolic blood pressure; SBP: systolic blood pressure; OM: office measurement; NPI: non-pharmacological intervention; PT: pharmacological treatment; HR: high risk; RF: risk factor; CVD: cardiovascular disease; DM: diabetes; CKD: renal damage; NR: not reported; ASCVD: atherosclerotic cardiovascular disease risk; LoE: level of evidence; CoR: class of recommendation (i.e., strength). In bold, throughout the table, the strength assigned to each recommendation when explicitly reported in the CPGs.

**Table 2:** World Health Organization (WHO) and International Society of Hypertension (ISH)—Overview of the included Clinical Practice Guidelines (CPGs).

Year and Edition	Ref.	HBP Definition, in mmHg	NPI Before PT	Treatment Threshold, in mmHg	Treatment Target, in mmHg	High-Risk Condition(s)	Quality and Strength of the Recommendations [Supporting Evidence]
1978 WHO	[27]	SBP ≥ 160 and/or DBP ≥ 95; borderline HBP: 140–160 SBP and/or 90–95 DBP	NA	SBP > 140 or DBP > 90	≤140/90	Not specifically addressed	NR
1983 WHO/ISH	[29]	DBP 90–105 (mild)	1–6 months	DBP > 100 after 1 month of NPI or DBP > 95 after 3 months of NPI	<90 DBP	Not specifically addressed	NR
1986 WHO/ISH	[30]	DBP 90–104 (mild)	1–6 months	DBP ≥ 100 after 1 month of NPI or DBP ≥ 95 after 3 months of NPI	<90 DBP	High SBP; age; history of CVD; renal disease; high cholesterol; familiar history of CVD	NR
1989 WHO/ISH	[31]	DBP 90–104 (mild)	1–6 months	DBP ≥ 100 after 3 months of NPI; DBP 95–99 after 3 months of NPI in presence of HR; DBP 90–94 after 6 months of NPI in presence of HR; DBP 95–99 after 6 months of NPI	<90 DBP and lower SBP if ≥160	SBP ≥ 160 mmHg; male gender; age; history of CVD; renal disease; familiar history of CVD; smoke	NR
1993 WHO/ISH	[32]	SBP 140–180 and/or DBP 90–105 (mild); ≥180 SBP and/or ≥105 DBP (moderate/severe)	3–6 months	SBP ≥ 140 or DBP ≥ 90 in presence of HR and for elderly subjects; SBP ≥ 160 or DBP ≥ 95 in absence of HR	Mild HBP and age < 60 y: target of 130–120/80; severe HBP and elderly subjects: <140/90	Male gender; age; history of CVD; renal disease; familiar history of CVD; smoke; cholesterol total and HDL; impaired fasting glucose	NR
1999 WHO/ISH	[33]	SBP ≥ 140 and/or DBP ≥ 90 with: mild (SBP 140–159/DBP 90–99; moderate: 160–179/100–109; severe: ≥180 and/or ≥110)	3–6 months	SBP ≥ 140 or DBP ≥ 90 after 3–6 months of NPI and medium risk; SBP ≥ 150 or DBP ≥ 95 after 6–12 months of NPI and low risk	<130 SBP/85 DBP in young, middle-aged and DM subjects; <140/90 in elderly subjects	Risk stratification based upon BP levels plus presence/absence of organ damage, smoke, dyslipidemia, DM, history of CVD (10-y risk of CVD: low: <15%; medium: 15–20%; high: 20–30%; very high: >30%)	NR
2003 WHO/ISH	[34]	SBP ≥ 140 and/or DBP ≥ 90	NA	SBP ≥ 140 or DBP ≥ 90, based upon risk category	<140 SBP; optimal: <130 SBP and <80 DBP	Risk stratification based upon BP levels plus presence/absence of organ damage, age, gender, smoke, dyslipidemia, DM, history of CVD (10-y risk of CVD: low: <15%; medium: 15–20%; high: ≥20%)	NR
2020 ISH	[28]	SBP ≥ 140 and/or DBP ≥ 90 (high-normal 130–139 SBP and/or 85–89)	3–6 months	SBP 140–159 or DBP 90–99 plus CVD/CKD/DM/OD	<140 SBP and <90 DBP; <65 y: <130 SBP and <80 DBP	Risk stratification based upon BP levels plus presence/absence of ≥1 RF (CVD history, organ damage, smoke, inactivity, DM, CKD)	Each recommendation classified as “Essential” or “Optimal”, but no explicit reference to supporting evidence
2021 WHO/ISH	[35]	SBP ≥ 140 and/or DBP ≥ 90	NA	SBP ≥ 140 or DBP ≥ 90; SBP 130–139 and CVD or DM or CKD or high CVD risk (**STRONG R**; Moderate-High LoE; **CONDITIONAL R** for DM or CKD or high CVD risk)	<140 SBP and <90 DBP (**STRONG R**; Moderate LoE); <130 SBP in the presence of CVD (**STRONG R**; Moderate LoE) or DM or CKD or high CVD risk (**CONDITIONAL R**; Moderate LoE)	Risk stratification based upon BP levels plus presence/absence of ≥1 RF (CVD history, organ damage, smoke, inactivity, DM, CKD)	Systematic literature review to include all the available evidence, rated through GRADE approach (high, moderate, low, very low Evidence—Strong vs. Weak or Conditional Recommendations)

HBP: hypertension; DBP: diastolic blood pressure; SBP: systolic blood pressure; NA: not assessed; NPI: non-pharmacological intervention; PT: pharmacological treatment; NR: not reported; HR: high risk; RF: risk factor; CVD: cardiovascular disease; DM: diabetes; CKD: renal damage; OD: organ damage; LoE: level of evidence. In bold, throughout the table, the strength assigned to each recommendation when explicitly reported in the CPGs.

**Table 3:** European Society of Hypertension and European Society of Cardiology (ESH/ESC)—Overview of the included Clinical Practice Guidelines (CPGs).

Year and Edition	Ref.	HBP Definition, in mmHg	NPI Before PT	Treatment Threshold, in mmHg	Treatment Target, in mmHg	High-Risk Condition(s)	Quality and Strength of the Recommendations [Supporting Evidence]
2003 ESH/ESC	[22]	SBP ≥ 140 or DBP ≥ 90 (high-normal 130–139 SBP or 85–89)	Based upon individual risk, but at least 3 months	SBP ≥ 180 and/or DBP ≥ 110; SBP 140–179 and/or DBP 90–109 and moderate risk; SBP ≥ 140 and/or DBP ≥ 90 after 3 months of NPI; SBP 130–139 and/or DBP 85–89 and very high/high risk	SBP < 140 and DBP < 90; for DM: SBP < 130 and/or DBP < 80	Risk stratification based on Framingham criteria or the SCORE Chart: CV risk low, moderate, high and very high based upon BP levels + *n*. of risk factors (male gender, smoke, dyslipidemia, obesity, familiar history of CVD, CRP), presence of OD or DM	NR
2007 ESH/ESC	[24]	SBP ≥ 140 and/or DBP ≥ 90 (high-normal 130–139 SBP and/or 85–89)	Based upon individual risk, no better specified than for “several weeks”. Start PT if unsuccessful	SBP ≥ 180 and/or DBP ≥ 110; SBP 140–179 and/or DBP 90–109 if ≥3 risk factors, or OD, or DM, or CKD; SBP ≥ 140 and/or DBP ≥ 90 after “several” months of NPI; SBP 120–139 and/or DBP 80–89 and established CVD or CKD	SBP < 140 and DBP < 90; for subjects with DM, stroke, myocardial infarction, proteinuria, or high/very high risk: SBP < 130 and/or DBP < 80	Risk stratification based upon the Framingham criteria or the SCORE Chart: CV risk low, moderate, high and very high based upon BP levels plus number of risk factors (RF) (male gender, smoke, dyslipidemia, obesity, familiar history of CVD, CRP), presence of OD or DM	NR
2013 ESH/ESC	[23]	SBP ≥ 140 and/or DBP ≥ 90 (high-normal 130–139 SBP and/or 85–89)	Based upon individual risk, no better specified than for “several weeks”. Start PT if unsuccessful	SBP ≥ 180 and/or DBP ≥ 110 (**I**); SBP 140–179 and/or DBP 90–109 if ≥3 risk factors, or OD, or DM, or CKD (**I**); SBP ≥ 140 and/or DBP ≥ 90 after “several” months of NPI (**II**); in individuals ≥ 80 y and SBP 140–159 and/or DBP 90–99, irrespective of risk category (**II**)	SBP < 140 and DBP < 90; for subjects with DM DBP < 85 (**ALL I**)	Risk stratification based upon the Framingham criteria or the SCORE Chart: CV risk low, moderate, high and very high according to SBP and DBP and number of RFs, OD, DM, CKD or symptomatic CVD	Strength of the recommendation (I to III) based upon the quality of the available evidence (A to C)
2018 ESH/ESC	[25]	SBP ≥ 140 and/or DBP ≥ 90 (high-normal 130–139 SBP and/or 85–89)	3–6 months in case of SBP 140–159 or DBP 90–99 and no CVD, CKD, or OD (low-moderate risk)	SBP ≥ 160 and/or DBP ≥ 100; SBP 140–159 and/or DBP 90–99 and CVD, CKD, or OD (**I**); SBP 140–159 and/or DBP 90–99 and low-moderate risk, after 3–6 months of NPI (**I**); SBP 130–139 and/or DBP 85–89 and CVD (coronary artery disease) (**II**)	in subjects < 65 y: SBP < 130; in subjects 65–79 y: SBP ≤ 130 and DBP ≤ 80; in subjects ≥ 80 y: SBP < 140 (**ALL I**)	Risk stratification based upon the SCORE Chart: CV risk low, moderate, high and very high according to SBP and DBP, number of RFs, and organ damage, DM, CKD or symptomatic CVD	Strength of the recommendation (I to III) based upon the quality of the available evidence (A to C) [SPRINT; ONTARGET trial; VALUE trial] [43,44,45]
2023 ESH	[26]	SBP ≥ 140 and/or DBP ≥ 90 (high-normal 130–139 SBP and/or 85–89)	3–6 months in case of SBP 140–149 and/or DBP 90–94 and no CVD, CKD, or OD (low-moderate risk)	SBP ≥ 140 and/or DBP ≥ 90 and symptomatic, or organ damage, or CKD, or established CVD (**I**); after unsuccessful 3–6 months of NPI in case of SBP 140–149 and/or DBP 90–94 and no CVD, CKD, or OD (**I**); ≥60 y: SBP ≥ 140 (any DBP) (**I**)	ideal: SBP < 130 and DBP< 80; 18–64 y: SBP < 130 and DBP < 80; 65–79 y: SBP < 140 and DBP < 80; ≥80 y: SBP 140–150 (**ALL I**)	Risk stratification based upon the SCORE2 Chart: CV risk low, moderate, high and very high according to sex, age, SBP, smoke, non-HDL cholesterol, CVD, DM, OD	Strength of the recommendation (I to III) based upon the quality of the available evidence (A to C)
2024 ESC	[6]	SBP ≥ 140 or DBP ≥ 90 (Elevated BP: SBP 120–139 or DBP: 70–89)	In case of SBP < 120 and DBP < 70 (normal); SBP 120–139 and DBP 70–89 (elevated BP) when: ≥85 y, moderate-to-severe frailty, symptomatic orthostatic hypotension, life expectancy < 3 y	SBP ≥ 140 and/or DBP ≥ 90 (**I**); SBP 120–139 and/or DBP 70–89 (excluding those in NPI) with 10-y CV risk ≥ 5% (**I**); SBP ≥ 130 and/or DBP ≥ 80 and CVD, CKD, DM, OD, familiar hypercholesterolemia, if NPI unsuccessful (**I**)	SBP < 130 (**I**) and/or DBP < 80 (**II**); SBP< 140 among: symptomatic subjects, frail subjects of any age, ≥85 y, lifespan < 3 y (**II**)	Risk stratification based upon the SCORE2 Chart: CV risk low, moderate, high and very high according to sex, age, SBP, smoke, non-HDL cholesterol, CVD, DM, OD	Strength of the recommendation (I to III) based upon the quality of the available evidence (A to C)

HBP: hypertension; DBP: diastolic blood pressure; SBP: systolic blood pressure; NPI: non-pharmacological intervention; NR: not reported; PT: pharmacological treatment; RF: risk factor; CVD: cardiovascular disease; DM: diabetes; CKD: renal damage; OD: organ damage. In bold, throughout the table, the strength assigned to each recommendation when explicitly reported in the CPGs.

**Table 4:** British Hypertension Society (BHS) and National Institute for Health and Care Excellence (NICE)—Overview of the included Clinical Practice Guidelines (CPGs).

Year and Edition	Ref.	HBP Definition, in mmHg	NPI Before PT	Treatment Threshold, in mmHg	Treatment Target, in mmHg	High-Risk Condition(s)	Quality and Strength of the Recommendations [Supporting Evidence]
1989 BHS I	[36]	NR (refer to DBP threshold)	NR. Active monitoring 3–6 m after initial BP assessment	DBP ≥ 100 after 3–4 months of active monitoring	Not addressed	Age, gender, CVD	NR
1993 BHS II	[41]	NR (refer to DBP threshold)	3–6 months	DBP ≥ 100; DBP ≥ 100 after 3–4 months of active monitoring; DBP 90–99 after 3–4 months of active monitoring if: >60 y or HR; among elderly: (a) SBP ≥ 160 and/or DBP ≥ 90 plus OD; (b) SBP 160–199 and DBP ≥ 95	DBP < 90; SBP < 160	OD, CVD history, DM, older age (>60 y), smoke, male gender, Col tot, familiar CVD	NR
1999 BHS III	[40]	SBP ≥ 140 and/or DBP ≥ 90 (high-normal 135–139 SBP and/or 85–89)	3–6 months	SBP ≥ 160 and/or DBP ≥ 100 (**A**); SBP ≥ 140 OR DBP ≥ 90 with DM (**B**); SBP 140–159 or DBP 90–99 with OD or DM or CVD complications or 10 y CHD risk ≥ 15% (**B**)	SBP < 140 and DBP < 90; SBP < 140 and DBP < 80 in DM (**A**)	Risk stratification based upon the Joint British Societies Cardiac Risk Assessor computer programme (CHD risk chart or Framingham risk chart): CVD history, ColTot, BP values, DM, OD	Strength of evidence rated using the North of England evidence-based Guidelines (I to IV evidence rating, and A to D strength of recommendations)
2004 BHS IV	[46]	SBP ≥ 140 and/or DBP ≥ 90 (high-normal 135–139 SBP and/or 85–89)	Up to 6 months	SBP ≥ 160 and/or DBP ≥ 100 (**A**); SBP140–159 and/or DBP 90–99 if DM, or OD or 10 y CVD risk ≥ 20% (**B**)	minimum acceptable: SBP < 150 and DBP < 90; non-DM: SBP < 140 and DBP < 85; DM: SBP < 130 and DBP < 80 (**B**)	Risk stratification based upon the Joint British Societies Cardiac Risk Assessor computer programme (CHD risk chart or Framingham risk chart): CVD history, ColTot, BP values, DM, OD	Strength of evidence rated using the North of England evidence-based Guidelines (I to IV evidence rating, and A to D strength of recommendations)
2004 NICE	[39]	SBP ≥ 140 and/or DBP ≥ 90	Up to 12 months	SBP ≥ 160 and/or DBP ≥ 100 (**A**); SBP > 140 and DBP > 90 if DM, or OD, or 10 y CVD risk ≥ 20% or CHD risk ≥ 15% or current CVD (**A**)	SBP ≤ 140 and/or DBP ≤ 90 (**A**)	Risk stratification based upon the Joint British Societies Cardiac Risk Assessor computer programme (CHD risk chart or Framingham risk chart): CVD history, ColTot, BP values, DM, OD	Strength of evidence rated using the AHRQ Classification (I to III evidence rating, and A to C strength of recommendations)
2011 NICE	[37]	SBP ≥ 140 and/or DBP ≥ 90	Based upon individual risk	SBP ≥ 150 and/or DBP ≥ 95; SBP140–159 and/or DBP 90–99 if OD, CVD, DM, CKD, 10 y CVD risk ≥20%	SBP < 140 and/or DBP < 90; for those ≥80 y: <150 and/or DBP < 90	Risk stratification based upon the Joint British Societies Cardiac Risk Assessor computer programme (CHD risk chart or Framingham risk chart): CVD history, ColTot, BP values, DM, OD	Quality of the supporting evidence rated as above, but overall rating of the strength of the recommendations not provided
2019 (upd. 2023) NICE	[38]	SBP ≥ 140 and/or DBP ≥ 90	Based upon individual risk (NICE CPG on CVD)	SBP ≥ 160 and/or DBP ≥ 100; subjects ≥ 80 y: SBP ≥ 150 and/or DBP ≥ 90; SBP140–159 and/or DBP 90–99 if 10 y CVD risk ≥ 20%	Subjects < 80 y: SBP < 140 and/or DBP < 90; SBP < 130 and/or DBP < 80 if DM or CKD. Subjects ≥ 80 y: SBP < 150 and/or DBP < 90; SBP < 140 and/or DBP < 90 if CKD; SBP < 130 and/or DBP < 80 if CKD plus ACR ≥ 70 mg/mmol	Risk stratification based upon the Joint British Societies Cardiac Risk Assessor computer programme (CHD risk chart or Framingham risk chart): CVD history, ColTot, BP values, DM, OD	GRADE approach for quality rating (high, moderate, low, very low LoE) and A to C strength of recommendations declared, but no details provided [HIVET] [47]

HBP: hypertension; DBP: diastolic blood pressure; SBP: systolic blood pressure; NR: not reported; upd.: updated; NPI: non-pharmacological intervention; PT: pharmacological treatment; HR: high risk; CHD: coronary heart disease; CVD: cardiovascular disease; DM: diabetes; CKD: renal damage; OD: organ damage; ColTot: total cholesterol; ACR: Albumin to creatinine ratio; LoE: level of evidence; AHRQ: Agency for Healthcare Research and Quality. In bold, throughout the table, the strength assigned to each recommendation when explicitly reported in the CPGs.

**Table 5:** Comparison of the currently accepted definitions, thresholds, targets, and grading approaches issued by each of the included organizations.

Issuing Organization	ACC/AHA	ESH/ESC	WHO/ISH	NICE
**Diagnostic thresholds (in mmHg)**	SBP ≥ 130 or DBP ≥ 80	SBP ≥ 140 or DBP ≥ 90	SBP ≥ 140 and/or DBP ≥ 90	SBP ≥ 140 and/or DBP ≥ 90
**Treatment thresholds (in mmHg)**	SBP > 130 or DBP > 80	SBP ≥ 140 and/or DBP ≥ 90	SBP ≥ 140 or DBP ≥ 90	SBP 140–159 and/or DBP 90–99 (10 y CVD risk ≥ 20%); always with SBP ≥ 160 and/or DBP ≥ 100
**Treatment targets (in mmHg)**	SBP ≤ 130 (encouraged < 120 SBP); DBP < 80 DBP (recommended for all individuals, irrespective of increased cardiovascular risk)	SBP < 130; DBP < 80	SBP < 140; DBP < 90	SBP < 140; DBP < 90
**Evidence-grading approach**	Grading scheme based upon Level of Evidence: (A) ≥1 RCT/meta-analysis; (B) 1 RCT/non-randomized studies; (C) expert opinion/standard of care and Class of Recommendation (I to III)	Grading scheme based upon Level of Evidence: (A) ≥1 RCT/meta-analysis; (B) 1 RCT/non-randomized studies; (C) expert opinion/standard of care and Class of Recommendation (I to III)	Certainty of evidence: High, Moderate, Low, Very Low according to GRADE approach. Evidence strength: Strong or Weak/Conditional based upon the Committee confidence that desirable effects of adhering to the recommendation outweighs undesirable effects	Grading scheme based upon Level of Evidence: (I) ≥ 1 RCT/meta-analysis; (II) non-randomized studies/quasi-experimental studies; (III) descriptive/case-control studies; (IV) expert opinion. Strength of recommendation classified from A to D

## Data Availability

The original contributions presented in this study are included in the article. Further inquiries can be directed to the corresponding author.
